# Psychometric performance of an assessment scale for strain in nursing care: The M-NCAS

**DOI:** 10.1186/1477-7525-2-62

**Published:** 2004-11-09

**Authors:** Leah Kleinman, Lori Frank, Gabrielle Ciesla, Marcia Rupnow, Henry Brodaty

**Affiliations:** 1 MEDTAP International, Inc., 2601 4^th ^Ave, Seattle, WA 98121, USA; 2 MEDTAP International, Inc., 7101 Wisconsin Avenue, Suite 600, Bethesda, MD 20814, (GC at the time the study was conducted, now at Medimmune), USA; 3 Janssen Pharmaceutica Inc., 1125 Trenton-Harbourton Road, Titusville, NJ 08560, USA; 4 School of Psychiatry, University of New South Wales, Randwick, New South Wales 2031, Australia

## Abstract

**Background:**

Multiple instruments exist to measure dementia behaviors, but the nursing staff perspective on those behaviors and their level of burden has not been well measured. The goal of this study was to examine the psychometric performance of the Modified Nursing Care Assessment Scale (M-NCAS), a 28-item nurse rating of burden associated with care for institutionalized individuals with dementia. Nurses rate items in terms of extent to which the behavior or characteristic is present ("attitude" domain), and extent to which it is a burden ("strain" domain).

**Methods:**

Data from 282 patients enrolled in a 12-week, double-blind, randomized clinical trial comparing risperidone treatment to placebo was used to evaluate M-NCAS item performance, internal consistency reliability, and construct validity. Empirical subscales were identified via exploratory factor analysis (EFA).

**Results:**

Four poorly-performing items were deleted from further analyses. EFA identified 3 "attitude" subscales and 5 "strain" subscales. Cronbach's alphas were 0.65 and above. Correlation with the Cohen-Mansfield Agitation Inventory and the BEHAVE-AD, clinical ratings of dementia behaviors, were low to moderate.

**Conclusion:**

The M-NCAS provides a valid and reliable means of obtaining care burden ratings from formal caregivers in long-term care, and provides a method for evaluating dementia interventions from the perspective of nursing staff.

## Background

Caring for the institutionalized dementia patient can be challenging for nursing staff [1,2]. Persons with dementia often exhibit such disturbing behaviors as pacing, use of inappropriate language, acting out, refusal of necessary care, hallucinations, and delusions. Nursing staff caring for persons with dementia may have difficulty forming relationships with their patients, and the stress associated with such care may contribute to staff turnover. Nursing staff turnover in nursing homes is high, ranging from 40%–96% annually in the United States [3]. The societal costs of nursing staff turnover (particularly in the current environment of nursing staff shortages) are great, and can be expected to increase as the number of institutionalized older adults increases. Studies of disturbing or distressing behaviors among institutionalized dementia patients demonstrate that these behaviors occur frequently, and are a source of great frustration to the nursing staff [4-9]. The prevalence of dementia in nursing homes, estimated to be close to 50% in the U.S. [10] and over 70% in Australia [11], coupled with the behaviors associated with dementia, combine to make caring for patients with dementia a particular challenge to nursing staff. Measuring this burden can provide another means of evaluating outcomes of dementia treatment in the nursing home setting.

There are measures relevant to the burden of paid/formal caregivers (i.e., nursing staff caring for dementia patients), but most focus on such overt behaviors as "forgetting what day it is" or "pull away" only (e.g., the Memory and Behaviors Problems Checklist [12] and the Resistiveness to Care Scale [2]). In their comprehensive examination of formal caregiver stress and cognitive impairment, Novak and Chappell [9] used an extensive individual face-to-face interview that lasted approximately one and a hours – a form of the Burden Interview [13] modified for institutional caregivers and Maslach's Burnout Inventory [14]. In their earlier work, they also asked 5 dementia-specific questions [15].

The Strain in Nursing Care Assessment Scale (NCAS) [9,10] was developed to address the need for a more comprehensive measure of nursing burden – specific to the long-term care setting – that includes sources of burden beyond overt behaviors. It is based on the conceptualization of burden as deriving from patient behaviors, as well as patient characteristics as perceived by the nurse carer. Unlike solely behavior-based measures completed by nurses, the NCAS addresses other aspects relevant to the provision of nursing care, such as nursing staff's perceptions regarding the meaningfulness of resident lives and the residents' level of gratefulness for care. The NCAS demonstrated the ability to capture changes in nurses' rating of difficulty regarding dementia patients' characteristics in a year-long study of a care intervention [16]. The Modified Strain in Nursing Care Assessment Scale (M-NCAS) was adapted from the NCAS developed and used in Swedish long-term care facilities [16,17]. The M-NCAS includes more items than the original instrument to capture the presence and impact of additional behaviors not represented in the original instrument, thus providing an even more comprehensive measure of nursing burden.

The M-NCAS was used in a clinical trial of an atypical antipsychotic, risperidone, for treatment of nursing home patients with behavioral and psychological signs and symptoms in dementia. This preliminary investigation describes the psychometric evaluation of the M-NCAS using data from the clinical trial; thus, it is a post-hoc assessment of reliability and validity. Assessment of psychometric properties included exploration of item performance, subscale assignment, and assessment of reliability and validity based on the available data from the clinical trial.

## Methods

### Design and Procedures

The psychometric evaluation used data from the baseline assessment of a multi-site, double-blind, placebo-controlled trial of risperidone in the treatment of behavioral and psychological symptoms among institutionalized patients with dementia. Fourteen sites throughout Australia and New Zealand recruited patients from the various nursing homes associated with each site. Patients were included in the clinical trial if they were 55 years of age or older, residing in a nursing home environment for one month, with a DSM-IV diagnosis of either dementia of the Alzheimer's type with behavioral disturbance, vascular dementia with behavioral disturbance, or mixed dementia, had scores less than or equal to 23 on the Mini-Mental Status Exam (MMSE) [18], and met aggressive item score criteria on the Cohen-Mansfield Agitation Inventory (CMAI) [19]. Aggressive item score criteria was defined as a score of at least 4 on one aggressive item, a frequency score of 3 on at least two aggressive items, or a frequency score of 2 on at least three aggressive items or two aggressive items occurring at a frequency of 2 and one at a frequency of 3 on the CMAI. These criteria were designed by the trial investigators to limit the sample to moderate to severe dementia patients having recognizable behavioral disturbances. Patients were required to have a carer who was able and committed to assisting the subject to comply with medication intake and the trial protocol, and who was willing to provide the information required at assessment interviews. Nursing home patients were excluded if they had medical or neurological conditions other than dementia which diminished cognitive function, dementia secondary to alcoholism, a diagnosis of major psychiatric comorbidity, substance abuse, tardive dyskinesia, clinically-uncontrolled medical conditions or laboratory abnormalities, a history of adult seizures, an administration of a depot injection or a long-acting neuroleptic within two treatment cycles of selection, hypersensitivity to neuroleptic treatment, or a history of failure to respond to risperidone of four weeks duration or participation in clinical trials with investigational drugs within the past four weeks. All patients or their appropriate representatives provided written informed consent, and the research protocol was approved by the appropriate institutional review board and state guardianship boards where required. Nurse carers were identified for each patient participant.

### Nursing Care Assessments

The Behavioral Pathology in Alzheimer's Disease Rating Scale (BEHAVE-AD), the CMAI, and the M-NCAS were administered to the nurse carers of the individual patients by the investigator or site coordinator at baseline, week 4, week 8, and week 12 (trial endpoints). Nurse carers could have more than one patient participating in the trial, and they completed questionnaires for each individual patient.

#### Modified Nursing Cares Assessment Scale (M-NCAS)

The M-NCAS is was adapted from the original NCAS instrument developed in Sweden, and contains 32 items based in part on the 21 original items of the NCAS. Additional items were selected based on comments made by long-term care nurses regarding their experience caring for dementia patients with dementia in long-term care facilities, and added to make the instrument more comprehensive.

The M-NCAS contains 32 items, with two domains per item: one addresses the occurrence and intensity of the behavior, in which the staff members indicate the extent to which they agree that the patient exhibits the behavior (the "attitude" domain); and one which addresses staff rating of the difficulty of coping with the behavior (the "strain" domain). Responses are measured on a four-point Likert-type scale (ranging from Agree to Don't Agree for the Attitude domain, and Very Easy to Very Difficult for the Strain domain). Lower scores are better for both domains. Domain total and subscale scores are calculated separately; total and subscale scores are calculated as an average of the individual item scores. The M-NCAS was translated from the original Swedish to English using standard forward and backward translation techniques.

For subscale analyses, if < 50% of the scale items were missing, the scale was scored with the mean score of the non-missing items for that individual used to impute a score for the missing items. If > 50% of the items were missing, no scale score was calculated; the subscale score was considered missing. Total scores were calculated only if < 50% of the items were missing. Missing item criteria was based on that recommended by Ware et al. [20].

#### Cohen-Mansfield Agitation Inventory (CMAI)

The CMAI is a 29-item scale specifically developed to assess agitated and disruptive behaviors, as well as care-related problems, occurring in demented subjects residing in nursing homes. The scale measures the frequency of behaviors, and systematically assesses agitation. The scale's 29 activities are rated on a 7-point scale indicating the frequency of a particular activity (range; never to a few times per hour). The activities are organized into 4 subscales: physical/aggressive, physical/non-aggressive, verbal/aggressive, and verbal/non-aggressive. The total aggression score is the sum of the physical and verbal aggression subscales. The total non-aggression score is the sum of the verbal and physical non-aggression subscales. A recall period of one week was used. The CMAI was used to assess the construct validity of the M-NCAS.

#### Behavioral Pathology in Alzheimer's Disease Rating Scale (BEHAVE-AD)

The BEHAVE-AD is a 25-item instrument designed to assess the severity of behavioral disturbances in subjects with dementia, based upon the caregiver's observation and report of the subject's behavioral problems. It consists of 7 subscales: paranoid and delusion ideation, hallucinations, activity disturbances, aggressiveness, diurnal rhythm disturbances, affective disturbances, and anxieties and phobias. Symptoms are rated on a scale from 0 (absence of the symptom) to 3 (greatest severity). A global item is included, in which a single judgment is made as to how troubling or dangerous the subject's behavior has been to the caregiver. A recall period of one month was used. The BEHAVE-AD was used to assess the construct validity of the M-NCAS.

### Data Analysis and Psychometric Evaluation

We evaluated the item performance, scaling characteristics, reliability, and construct validity of the M-NCAS using baseline assessment data.

#### Item Performance and Scaling Characteristics

Item performance was examined to identify the presence of items that may reduce the instrument's ability to detect changes over time, and to discriminate between groups. The characteristics of individual M-NCAS items examined were mean, minimum and maximum responses, percent missing, floor and ceiling effects, and item-total correlations. These data were used to determine if some items should be deleted for subsequent analyses (e.g., item-to-total correlations below 0.40 [21]). Exploratory factor analysis was performed to evaluate the underlying subscale structure of the M-NCAS.

#### Reliability

Internal consistency reliabilities of total and subscale scores were estimated using Cronbach's coefficient α. Data to examine test-retest reliability was not available.

#### Construct Validity

Correlations were determined for total and subscale scores for attitude and strain domains and the CMAI physical and verbal aggression and non-aggression subscales. Correlations were also calculated between M-NCAS scores and the total score, seven subscales, psychotic symptoms subtotal and global rating question of the BEHAVE-AD, and the CGI-S and CGI-C scores. Pearson correlations were used unless otherwise specified. Correlations were expected to be low to moderate (0.20 ≤ r < 0.40).

All statistical analyses were conducted using SAS statistical software version 8.0. A significance level of 0.05 and 2-tailed tests were used unless otherwise noted. No adjustments were made for multiple comparisons.

## Results

Two hundred and eighty-two patients had evaluable baseline data for the psychometric evaluation. The majority of patients were female (72.4%) and Caucasian (98.4%); 11.5% were between the ages of 65 and 74, while 87.1% were over age 74. Most subjects had a diagnosis of Alzheimer's Disease (59.1%), while 28.3% had vascular dementia and 12.5% had mixed dementia.

Nurse carers were primarily Caucasian. Further demographic data and information about the number and type of carers were not available.

### Item Performance

Items and their distributional characteristics were reviewed. See Table 1 for a listing of items, mean scores, and floor (% of responses at 1) and ceiling effects (% of responses at 4). The attitude domain contained several items with high floor and ceiling effects. Item 12 had 79% of responses at floor – the highest among all items. Items 19 and 28 had the highest percentages of responses at ceiling (76% and 77%, respectively). Only one person had any missing data at the baseline visit. Examination of item correlation to total and subscale scores on the attitude domain demonstrated low or inverse correlations for four items (Item 5: tries to influence others in order to maintain control of his/her life; Item 12: is submissive; Item 27: patterns of behaviors you can foresee; and Item 19: has little control over his/her difficult behavior) (data not shown).

**Table 1:** Items in the M-NCAS

		**Attitude**			**Strain**		
			
	**Description**	**Mean (SD)**	**Floor (N, %)**	**Ceiling (N, %)**	**Mean (SD)**	**Floor (N, %)**	**Ceiling (N, %)**
Item 1^R^	Seems to behave in a completely aimless way	3.16 (1.08)	1 (45, 15.96%)	4 (144, 51.06%)	2.64 (0.93)	1 (39, 13.83%)	4 (50, 17.73%)
Item 2^R^	Is anxious	3.3 (0.97)	1 (28, 9.93%)	4 (160, 56.74%)	2.79 (0.88)	1 (26, 13.83%)	4 (59, 20.92)
Item 3^R^	Is unpredictable	3.2 (1.1)	1 (50, 50.0%)	4 (169, 59.9%)	2.86 (0.92)	1 (26, 9.22%)	4 (74, 26.24%)
Item 4	Does things for a reason	2.86 (1.19)	1 (56, 45.0%)	4 (127, 45.0%)	2.57 (0.96)	1 (49, 17.38%)	4 (47, 16.67%)
*Item 5*^1^	*Tries to influence others in order to maintain control of his/her own life*	*2.904 (1.3)*	*1 (74, 26.24%)*	*4 (154, 54.6%)*	*2.22 (1.06)*	*1 (92, 32.62%)*	*4 (41, 14.54%)*
Item 6	Is calm	3.17 (1.04)	1 (13, 4.61%)	4 (165, 58.5%)	2.76 (0.92)	1 (28, 9.93%)	4 (64, 22.70%)
Item 7^R^	Is apathetic/seems to have limited emotions	2.54 (1.32)	1 (109, 38.65%)	4 (101, 35.82%)	2.48 (0.92)	1 (47, 16.67%)	4 (37, 13.12%)
Item 8^R^	Is selfish	2.40 (1.32)	1 (116, 41.41%)	4 (94, 33.33%)	2.32 (0.99)	1 (69, 24.47%)	4 (39, 13.83%)
Item 9	Is rewarding to work with	2.37 (1.20)	1 (87, 30.85%)	4 (82, 29.08%)	2.55 (0.97)	1 (46, 16.31%)	4 (51, 18.09%)
Item 10	Is grateful for what is done for him/her	2.49 (1.18)	1 (71, 25.18%)	4 (89, 31.56%)	2.30 (0.94)	1 (61, 21.63%)	4 (33, 11.70%)
Item 11^R^	Is paranoid	2.40 (1.29)	1 (111, 39.36%)	4 (89, 31.56%)	2.43 (1.04)	1 (65, 23.05%)	4 (52, 18.44%)
*Item 12*^*R*^	*Submissive/gives in to everything done to him/her*	*1.47 (0.93)*	*1 (222, 78.72%)*	*4 (15, 5.32%)*	*2.97 (0.89)*	*1 (20, 7.09%)*	*4 (88, 31.21%)*
Item 13^R^	Is attention seeking	2.57 (1.31)	1 (101, 38.82%)	4 (105, 37.23)	2.48 (1.00)	1 (56, 19.86%)	4 49, 17.38%)
Item 14^R^	Is manipulative	1.97 (1.21)	1 (158, 56.03%)	4 (51, 18.09)	2.16 (0.96)	1 (82, 29.08%)	4 (29, 10.28%)
Item 15^R^	Is ungrateful for the care he/she receives	2.23 (1.18)	1 (113, 40.07%)	4 (57, 20.21%)	2.30 (0.96)	1 (66, 23.40%)	4 (35, 12.41%)
Item 16^R^	Is frightened/vulnerable	2.77 (1.18)	1 (72, 25.53%)	4 (109, 38.65%)	2.56 (0.90)	1 (38, 13.48%)	4 (41, 14.54%)
Item 17^R^	Is lonely	2.76 (1.20)	1 (65, 23.05%)	4 (112, 39.72%)	2.49 (0.87)	1 (40, 14.18%)	4 (32, 11.35%)
Item 18^R^	Has to concentrate exclusively on his/her needs in order to survive	2.36 (1.30)	1 (115, 40.93%)	4 (90, 32.03%)	2.40 (0.96)	1 (58, 20.64%)	4 (38, 13.52%)
*Item 19*^*R*^	*Has little control over his/her difficult behavior*	*3.59 (1.30)*	*1 (14, 4.97%)*	*4 (214, 75.89%)*	*3.16 (0.77)*	*1 (10, 3.55%)*	*4 (100, 35.46%)*
Item 20^R^	Is deliberately difficult	2.06 (1.17)	1 (138, 48.94%)	4 (46, 16.31%)	2.58 (1.00)	1 (52, 18.44%)	4 (53, 18.79%)
Item 21	Tries to maintain some independence	2.33 (1.27)	1 (108, 38.30%)	4 (89, 31.56%)	2.56 (0.83)	1 (34, 12.06%)	4 (29, 10.28%)
Item 22	Knows what he/she wants and stands up for his/herself	2.30 (1.25)	1 (103, 36.53%)	4 (86, 30.50%)	2.79 (0.85)	1 (28, 9.93%)	4 (51, 18.09%)
Item 23	Seems to experience the normal range of emotions	2.83 (1.24)	1 (63, 22.34%)	4 (134, 47.52%)	2.59 (0.83)	1 (30, 10.64%)	4 (33, 11.70%)
Item 24	Friendly	2.05 (1.03)	1 (94, 33.33%)	4 (48, 17.02%)	2.21 (0.84)	1 (58, 20.57%)	4 (17, 6.03%)
Item 25^R^	Needs someone close by all the time/is demanding	2.57 (1.31)	1 (104, 36.88%)	4 (101, 35.82%)	2.58 (0.96)	1 (43, 15.25%)	4 (53, 18.79%)
Item 26^R^	Has an empty life	2.81 (1.17)	1 (60, 21.28%)	4 (110, 39.01%)	2.56 (0.92)	1 (41, 14.54%)	4 (42, 14.89%)
*Item 27*^*R*^	*Has patterns of behavior you can foresee*	*3.00 (1.12)*	*1 (53, 18.79%)*	*4 (125, 44.33%)*	*2.75 (0.85)*	*1 (24, 8.51%)*	*4 (50, 17.73%)*
Item 28^R^	Is stubborn/resistive	3.68 (0.71)	1 (12, 4.26%)	4 (218, 77.31%)	3.22 (0.78)	1 (12, 4.26%)	4 (113, 40.07%)
Item 29^R^	Is aggressive/ hostile	3.44 (0.91)	1 (26, 9.22%)	4 (181, 64.18%)	3.18 (0.84)	1 (16, 5.67%)	4 (113, 40.07%)
Item 30	Has a meaningful life	3.17 (1.00)	1 (25, 8.87%)	4 (145, 53.19%)	2.59 (0.86)	1 (32, 11.35%)	4 (38, 13.48%)
Item 31	Compliant/voluntarily co-operative	3.10 (1.01)	1 (9, 3.19%)	4 (150, 53.19%)	2.87 (0.80)	1 (16, 5.67%)	4 (58, 20.57%)
Item 32^R^	Gives no job satisfaction	2.49 (0.91)	1 (43, 15.25%)	4 (37, 13.12%)	2.49 (0.91)	1 (43, 15.25%)	4 (37, 13.12%)

^1^*Italics indicate items deleted from instrument in subsequent analyses*. ^*R*^*Item is reverse-coded for the attitude total and subscale scores. Attitude ranges from 1 (agree) to 4 (don't agree) and Strain ranges from 1 (very easy) to 4 (very difficult) Lower scores are considered better for both dimensions*.

Examination of any floor or ceiling effects on the strain domain demonstrated no outliers. Item-to-item correlations appeared acceptable, as did item-to-total correlations, all generally above 0.30.

### Factor Analysis

With the number of factors unspecified, no clear factor pattern was discernible. Next, a six-factor solution for both dimensions (strain and attitude) was examined, based on the suitability of six factors for the original instrument [16]. Examination of the oblique rotated factor loadings demonstrated substantial item overlap. Results of subsequent factor analyses indicated that the best solution was a three-factor solution for the attitude domain, and a five-factor solution for the strain domain. Examination of the three-factor solution for the attitude dimension indicated that three items (item 19 has little control over his/her difficult behavior, item 27 has patterns of behavior you can foresee, item 7 is apathetic/seems to have limited emotions) did not perform well on the factor analysis – either overlapping substantially with another factor, or producing factor loadings less than 0.30.

Based on overall item analysis results, including floor and ceiling effects, item-to-item and item-to-total correlation results, and a review of factor analysis results, four poorly-performing items were deleted from the attitude domain: item 5 tries to influence others in order to maintain control of his/her own life; item 12 submissive/gives in to everything done to him/her; item 19 has little control over his/her difficult behavior; and item 27 has patterns of behavior you can foresee. The removal of these items resulted in three distinct subscales for the attitude domain, demonstrating good approximation of simple structure. The three factors, all with variable loadings of 0.30 or above were "Attention-seeking" (e.g., "is manipulative", "needs someone close by all the time/is demanding"), "Autonomy" (e.g., "does things for a reason", "is apathetic/seems to have limited emotions"), and "Difficulty" (e.g. "is unpredictable" and "is friendly"). Items were reverse-coded as appropriate.

To ensure inter-domain consistency, we removed the same four items from the strain dimension, thus resulting in an acceptable five-factor solution for this domain. The five factors, all with variable loadings of 0.30 or above, are "Affect" (e.g., "is calm", 'is anxious"), "Job satisfaction" (e.g., "is grateful for what is done for him/her", "gives no job satisfaction"), "Neediness" (e.g., "is selfish", "is manipulative"), "Predictability" (e.g., "knows what he/she wants and stands up for his/herself", "is aggressive/resistive"), and "Self-direction" (e.g., "is frightened/vulnerable", "seems to experience the normal range of emotions"). See Tables 2 and 3 for a list of specific items in each domain.

**Table 2:** Item-to-subscale correlations for the attitude domain of the M-NCAS (28 items) at baseline

**Item**		**Attention Seeking**	**Autonomy**	**Difficulty**
Item 2:	Is anxious	0.573	0.073*	0.117
Item 6:	Is calm	0.492	0.194	0.338
Item 11:	Is paranoid	0.569*	-0.059	0.288
Item 13:	Is attention seeking	0.673	-0.203	0.174
Item 14:	Is manipulative	0.588	-0.199	0.343
Item 16:	Is frightened/vulnerable	0.425	0.124	-0.078*
Item 17:	Is lonely	0.568*	0.074	-0.009*
Item 18:	Has to concentrate exclusively on his/her own needs in order to survive	0.546	-0.167	0.256
Item 25:	Needs someone close by/is demanding	0.706	-0.041*	0.129
Item 26:	Has an empty life	0.462	0.305	0.286
Item 1:	Seems to behave in a completely aimless way	0.089*	0.479	0.0750*
Item 4:	Does things for a reason	-0.084	0.615	0.0043
Item 7:	Is apathetic/seems to have limited emotions	0.037	0.514*	0.222
Item 21:	Tries to maintain some independence	0.020*	0.652	0.115*
Item 22:	Knows what he/she wants and stands up for his/herself	-0.141	0.696	-0.013*
Item 23:	Seems to experience the normal range of emotions	-0.044*	0.601	-0.194
Item 30:	Has a meaningful life	0.170	0.397	0.187
Item 3:	Is unpredictable	0.164	0.082*	0.387
Item 8:	Is selfish	0.391*	-0.073	0.523
Item 9:	Is rewarding to work with	0.159	0.241	0.651
Item 10:	Is grateful for what is done for him/her	0.010	0.326*	0.630
Item 15:	Is ungrateful for the care he/she receives	0.279	0.127	0.656
Item 20:	Is deliberately difficult	0.259	-0.071*	0.482
Item 24:	Friendly	0.076*	0.251	0.628
Item 28:	Is stubborn/resistive	0.090*	-0.012*	0.482
Item 29:	Is aggressive/hostile	0.114*	0.023*	0.533
Item 31:	Compliant/voluntarily co-operative	0.172	0.225	0.595
Item 32:	Gives no job satisfaction	0.196	0.066*	0.525

*All correlations significant at p < 0.05, except as indicated

**Table 3:** Item-to-subscale correlations for the strain domain of the M-NCAS (28 items) at baseline

**Item**		**Affect**	**Job Satisfaction**	**Neediness**	**Predictability**	**Self Direction**
Item 1:	Seems to behave in a completely aimless way	0.727	0.447	0.409	0.348	0.488
Item 2:	Is anxious	0.705	0.393	0.472	0.397	0.567
Item 3:	Is unpredictable	0.694	0.441	0.479	0.452	0.306
Item 4:	Does things for a reason	0.675	0.378	0.407	0.462	0.347
Item 6:	Is calm	0.715	0.541	0.445	0.538	0.393
Item 7:	Is apathetic/seems to have limited emotions	0.702	0.533	0.444	0.444	0.539
Item 9:	Is rewarding to work with	0.572	0.777	0.475	0.536	0.422
Item 10:	Is grateful for what is done for him/her	0.523	0.840	0.560	0.490	0.516
Item 15:	Is ungrateful for the care he/she receives	0.520	0.828	0.601	0.521	0.513
Item 24:	Friendly	0.406	0.728	0.477	0.500	0.515
Item 32:	Gives no job satisfaction	0.549	0.807	0.536	0.580	0.615
Item 8:	Is selfish	0.485	0.560	0.764	0.467	0.386
Item 11:	Is paranoid	0.428	0.447	0.627	0.419	0.387
Item 13:	Is attention seeking	0.483	0.432	0.809	0.389	0.471
Item 14:	Is manipulative	0.442	0.482	0.784	0.389	0.381
Item 18:	Has to concentrate exclusively on his/her own needs in order to survive	0.504	0.497	0.753	0.465	0.592
Item 20:	Is deliberately difficult	0.414	0.510	0.672	0.537	0.450
Item 25:	Needs someone close by all the time/is demanding	0.469	0.483	0.717	0.450	0.597
Item 21:	Tries to maintain some independence	0.476	0.477	0.495	0.738	0.494
Item 22:	Knows what he/she wants and stands up for his/herself	0.433	0.485	0.540	0.773	0.455
Item 28:	Is stubborn/resistive	0.515	0.498	0.452	0.815	0.383
Item 29:	Is aggressive/hostile	0.487	0.510	0.436	0.823	0.358
Item 31:	Compliant/voluntarily co-operative	0.557	0.623	0.464	0.780	0.525
Item 16:	Is frightened/vulnerable	0.476	0.423	0.413	0.383	0.710
Item 17:	Is lonely	0.518	0.489	0.550	0.456	0.803
Item 23:	Seems to experience the normal range of emotions	0.532	0.536	0.457	0.537	0.725
Item 26:	Has an empty life	0.445	0.557	0.581	0.396	0.806
Item 30:	Has a meaningful life	0.429	0.476	0.435	0.403	0.793

Following the removal of these 4 items, item-to-subscale correlations were examined for all subscales. In all cases, items correlated moderately to highly with the subscales to which they were assigned via the factor analysis. See Tables 2 and 3.

### Reliability

The internal consistency reliability (Cronbach's alpha) for the attitude and strain total scores was good – 0.79 and 0.95, respectively. Internal consistency reliability for subscales in both domains was excellent in general. See Table 4.

**Table 4:** Internal consistency reliability

**Scale**	**Cronbach's α**
**Attitude Domain**	
Total Score	0.790
Autonomy	0.646
Attention Seeking	0.759
Difficulty	0.776
**Strain Domain**	
Total Score	0.945
Affect	0.795
Job Satisfaction	0.856
Neediness	0.856
Predictability	0.845
Self Direction	0.826

### Construct Validity

Table 5 summarizes the correlations between the M-NCAS attitude total and subscale scores and the CMAI total aggression, total non-aggression, and subscale scores. In general, correlations were low to moderate [22]. The highest correlation was between attention-seeking and verbal non-aggression (r = 0.68; p < 0.01).

**Table 5:** Correlations^1 ^between the CMAI and the M-NCAS attitude and strain domains

	**CMAI Scales**					
	
**M-NCAS Scales**	Physical Aggression	Physical Non-Aggression	Verbal Aggression	Verbal Non-Aggression	Total Aggression	Total Non-Aggression
**Attitude Domain**						
Total Score	0.286*	0.079	0.325*	0.467*	0.336*	0.306*
Attention Seeking	0.060	0.142	0.202 *	0.675*	0.107	0.463*
Autonomy	0.145	0.011	0.016	-0.074	0.129	-0.031
Difficulty	0.370*	-0.005	0.390*	0.242*	0.426*	0.125
**Strain Domain**						
Total Score	0.290*	0.141	0.273*	0.344*	0.324*	0.286*
Affect	0.330*	0.211	0.193*	0.246*	0.337*	0.286*
Job Satisfaction	0.320*	0.0189	0.333*	0.210	0.367*	0.125
Neediness	0.150*	0.128	0.222	0.458*	0.190*	0.338*
Predictability	0.297*	0.166*	0.258*	0.154*	0.327*	0.204
Self Direction	0.151*	0.060	0.143	0.302*	0.170*	0.204*

*significant at p < 0.01; ^1^Pearson product-moment correlations

Table 5 also summarizes the correlations between the strain total and subscale scores and the CMAI total aggression, total non-aggression, and subscale scores. Correlations were generally low to moderate, with only one correlation exceeding 0.40 (verbal non-aggression and neediness; r = 0.46, p < 0.01).

Table 6 presents the correlations between the M-NCAS attitude total and subscale scores and the BEHAVE-AD. With the exception of the activity disturbance subscale, most correlations between the BEHAVE-AD subscales and the attitude total score were statistically significant. In general, the attention-seeking attitude subscale was related to BEHAVE-AD scores, but the autonomy subscale was not. Results were mixed for the difficulty subscale, with a correlation of 0.47 to the aggressiveness BEHAVE-AD subscale.

**Table 6:** Correlations^1 ^between the BEHAVE-AD and the M-NCAS

	**BEHAVE-AD Scales**							
	
**M-NCAS Scales**	Total BEHAVE-AD	Paranoid and delusional ideation	Hallucination	Activity disturbance	Aggressiveness	Diurnal rhythm disturbance	Affective disturbance	Anxiety and phobias
**Attitude Domain**								
Total Score	0.380*	0.272*	0.159*	0.078	0.386*	0.182*	0.225*	0.354*
Attention Seeking	0.478*	0.421*	0.158*	0.091*	0.269*	0.185*	0.373*	0.556*
Autonomy	-0.059	-0.176*	0.044	0.021	-0.032	0.101	-0.015	-0.041
Difficulty	0.268*	0.207*	0.104	0.038	0.471*	0.079	0.053	0.128
**Strain Domain**								
Total Score	0.397*	0.281*	0.134*	0.146*	0.425*	0.209*	0.156*	0.353*
Affect	0.360*	0.195*	0.137	0.243*	0.362*	0.191*	0.169	0.286*
Job Satisfaction	0.265*	0.161*	0.074	0.061	0.431*	0.121	0.061	0.210*
Neediness	0.383*	0.325*	0.140	0.084	0.327*	0.234*	0.165*	0.372*
Predictability	0.331*	0.246*	0.107	0.169*	0.437	0.144	0.028	0.217*
Self Direction	0.310*	0.224*	0.093	0.066	0.241*	0.164*	0.213*	0.369*

*p < 0.01; ^1^Pearson product-moment correlations

Table 6 also presents results for the M-NCAS strain domain and BEHAVE-AD score correlations. The total and most of the strain subscale scores were significantly related to BEHAVE-AD total and subscale scores.

## Discussion

The M-NCAS demonstrated good psychometric properties based on an analysis of baseline data collected during a clinical trial of risperidone versus placebo, as reported in this preliminary investigation. Item performance, particularly floor and ceiling effects (together with item-total correlation data), suggested that four items on the attitude domain were performing poorly from a psychometric standpoint. Two items performed poorly on item correlations as well as factor analysis, while item 12 had a high floor effect as well as poor correlations. The remaining 28 items still capture sufficiently comprehensive data on the domains of interest, based on content review and on the empirical performance of subscales derived from them. The collection of additional data would enhance the confidence of conclusions regarding M-NCAS performance.

In general, the M-NCAS demonstrated excellent internal consistency reliability, with only the autonomy subscale producing a Cronbach's alpha below 0.70. Cronbach's alpha values of 0.70 or greater are considered suitable for use in the analyses of group comparisons. Supporting construct validity of the M-NCAS were the moderate and significant correlations to the CMAI and BEHAVE-AD. Correlation with these measures was not expected to be high, given the more expansive focus of the M-NCAS relative to the CMAI and BEHAVE-AD.

These results support the use of the M-NCAS for the collection of valid, reliable, and comprehensive information on the burden experienced by nurse carers when caring for dementia patients in long-term care settings. The instrument can assist in staff management by identifying nurse carers with the greatest levels of burden – ideally so that remedial action could be taken, either through extra support for staff members or through the shifting of caseloads from particularly difficult patients for nurses at risk for attrition.

There are several limitations to this study. Of major concern is the lack of information on the nurse carers themselves. No data was available as to the type of carer, the duration they had cared for the patient, and their sociodemographic characteristics. The inability to characterize the respondents to the M-NCAS is a limitation in interpreting generalizability of results. No control was attempted for nurse raters, so the possibility exists that measurement properties of the M-NCAS differed across nurse raters – therefore skewing the results. The extent to which rater effects limit the validity of results is likely to be minimal, however, given that multiple subjects were being rated by each nurse rater. Future examination of between-subject properties would be helpful. The sample size for the study is small, relative to the standard recommendations regarding the number of subjects per item for factor analysis. However, we set criteria for relatively moderate to high factor loadings. Of note is that the final recommendations for the measure length are closer to that guideline. Because this was a post-hoc analysis of clinical trial data, the instruments selected for use in assessing construct validity may not have been optimal, in that they did not assess nursing strain per se. However, they did assess the propensity of exhibiting certain behaviors – as does the M-NCAS. Additional data on stability across time (test-retest reliability) is desirable for a full psychometric description of the M-NCAS, and was not available from this study. Finally, this sample of nurse carers was almost exclusively Caucasian. Therefore, the generalizability of these results to nurse carers of different ethnicities, and in different locales is limited.

## Conclusions

The M-NCAS enables the detection of the presence or absence of specific behaviors similar to checklists (the attitude scale), but extends that information by providing a rating of the degree of burden of each aspect rated. It possesses good psychometric properties for use with nurse carers working with Alzheimer's patients in long-term care facilities. The M-NCAS provides a unique approach to identifying both positive and negative behaviors, and to quantifying the amount of stress felt by carers as a result of these behaviors. The nursing staff perspective on residents with dementia is unique [23], and the M-NCAS exploits this perspective by capturing the aspects of residents beyond overt behaviors.

## List of Abbreviations

Behavioral Pathology in Alzheimer's Disease Rating Scale (BEHAVE-AD)

Cohen-Mansfield Agitation Inventory (CMAI)

Exploratory Factor Analysis (EFA)

Mini-Mental Status Exam (MMSE)

Modified Strain in Nursing Care Assessment Scale (M-NCAS)

Strain in Nursing Care Assessment Scale (NCAS)

## Authors' contributions

LK participated in the data analysis design, data interpretation, and drafting of the manuscript. LF participated in the design, data interpretation, and drafting of the manuscript. GC performed the statistical analyses. MR conceived of the study and participated in data interpretation. HB conceived of the study and participated in both data collection and interpretation. All authors read and approved the final manuscript.
